# Factors Associated With Deep Bite Severity in Children With Primary and Mixed Dentition: A Cross-Sectional Epidemiological Study

**DOI:** 10.7759/cureus.113863

**Published:** 2026-08-03

**Authors:** Mekala Sirisha, Akshay Goje, Shyamalima Bhattacharyya, Anitha Jolige, Khaja Raziuddin Ansari, Hareesh Veeramalla, Tejaswi Kala

**Affiliations:** 1 Department of Public Health Dentistry, Tirumala Institute of Dental Sciences and Research Center, Nizamabad, IND; 2 Department of Orthodontics, Sri Sai College of Dental Surgery and Hospital, Vikarabad, IND; 3 Department of Public Health Dentistry, Regional Dental College, Guwahati, IND; 4 Department of Pedodontics and Preventive Dentistry, Nanded Rural Dental College and Research Center, Nanded, IND; 5 Department of Dentistry, Kalyani Dental Hospital, Begumpet, IND; 6 Department of Oral and Maxillofacial Surgery, Government Medical college, Mulugu, IND

**Keywords:** child, cross-sectional studies, deep bite, malocclusion, mixed dentition

## Abstract

Introduction: Deep bite is one of the most common vertical malocclusions encountered during childhood and may adversely affect oral function, periodontal health, facial esthetics, and long-term occlusal stability. This study aimed to evaluate the severity of deep bites and identify the demographic, extraoral, and intraoral factors associated with deep bite severity among children aged 5-12 years. Materials and methods: A cross-sectional study was conducted among 1,400 school-going children aged 5-12 years with primary and mixed dentition. The participants were selected using a two-stage proportionate cluster random sampling technique. Deep bite severity was assessed using a study-specific modified four-grade clinical classification system. Demographic characteristics, oral habits, facial profile, body build, lower facial height, lip competence, mentolabial sulcus, upper labial frenal attachment, molar relationship, and overjet were also recorded. Associations were evaluated using the chi-square test and Cramér's V test, and cumulative odds ordinal logistic regression was performed to identify independent predictors of deep bite severity. Statistical significance was set at p < 0.05. Results: Among the 1,400 children evaluated, grade 1 deep bite was observed in 734 children (52.4%), grade 2 in 337 children (24.1%), grade 3 in 259 children (18.5%), and grade 4 in 70 children (5.0%). Significant associations were observed between deep bite severity and oral habits, body build, facial profile, lower facial height, lip competence, mentolabial sulcus, and molar relationship (all p < 0.001). In contrast, upper labial frenal attachment was not (p = 0.341). Ordinal logistic regression identified athletic body build (odds ratio (OR) = 6.505, p = 0.003), decreased lower facial height (OR = 9.966, p < 0.001), increased lower facial height (OR = 3.362, p = 0.006), and increased overjet (OR = 1.255, p = 0.013) as significant independent predictors of greater deep bite severity. Conclusions: Deep bite severity was significantly associated with oral habits, craniofacial morphology, lower facial height, lip competence, mentolabial sulcus, and molar relationships. Athletic body build, altered lower facial height, and increased overjet were identified as independent predictors of greater deep bite severity, highlighting the importance of early clinical assessment for identifying children at risk of greater observed severity.

## Introduction

Malocclusion is one of the most prevalent oral health problems affecting children worldwide. It is considered a significant public health concern because of its impact on oral function, facial aesthetics, psychosocial well-being, and quality of life. Among the various occlusal discrepancies, a deep bite is one of the most frequently encountered vertical malocclusions [1,2]. It is characterized by an excessive vertical overlap of the maxillary incisors over the mandibular incisors and may arise from a combination of skeletal, dentoalveolar, genetic, and environmental factors. If left untreated during childhood, a deep bite may predispose individuals to abnormal tooth wear, periodontal trauma, and functional disturbances. It may be associated with temporomandibular disorders and compromised facial esthetics [3,4].

The development of a deep bite is multifactorial. It has been associated with facial growth patterns, lower facial height, molar relationships, incisor inclination, oral habits such as thumb sucking and tongue thrusting, lip incompetence, and altered craniofacial morphology [5,6]. Since these factors are established during childhood, early identification of at-risk children provides an opportunity for interceptive orthodontic treatment, thereby minimizing the complexity of future comprehensive orthodontic care.

Although several epidemiological studies have reported the occurrence of malocclusion in different populations, there is limited information regarding the severity of deep bite and the factors associated with its clinical presentation among Indian children, particularly those residing in rural and semi-urban communities [6,7]. Region-specific epidemiological data are essential for understanding the distribution and severity of deep bite, identifying children at a greater risk of severe malocclusion, and planning preventive and interceptive orthodontic services. Therefore, the present cross-sectional epidemiological study was undertaken to evaluate the distribution and severity of deep bite among children aged 5-12 years with primary and mixed dentition. The primary objective was to assess the prevalence of different deep bite severity grades. The secondary objective was to evaluate the associations of deep bite severity with demographic characteristics, oral habits, facial morphology, and extraoral and intraoral clinical findings using cumulative odds ordinal logistic regression analysis.

## Materials and methods

Study design, setting, location, duration, and ethical approval

This cross-sectional study was conducted in the Department of Public Health Dentistry, Tirumala Institute of Dental Sciences and Research Center, Nizamabad, India, among school-going children aged 5-12 years with primary and mixed dentition. The study was conducted between November 2024 and December 2025, following approval from the Institutional Ethics Committee (approval number: TIDSRC/233/2024, dated November 18, 2024). Permission was obtained from the concerned educational authorities and school administrations before commencement of the survey. Written informed consent was obtained from parents or legal guardians, while verbal assent was obtained from the participating children before the clinical examination.

Sample size estimation

Before the initiation of the main survey, a pilot study involving 183 children aged 5-12 years was conducted to assess the feasibility of the study protocol, evaluate examiner calibration, identify practical challenges during field data collection, and estimate the proportion of children with deep bites for sample size calculation. Because no published epidemiological data specific to the target population were available, the prevalence estimate used for sample size determination was derived from this pilot study. The pilot study demonstrated that 38% of the examined children exhibited deep bites; this estimate was subsequently used for sample size determination. The required sample size was calculated using the formula

\[ n = \frac{Z^{2} \times P \times q}{d^{2}} \]

assuming a 95% confidence level (Z = 1.96), expected proportion (p) of 38%, q = 62%, and absolute precision of 3.5%. After incorporating a design effect of 1.5 to account for cluster sampling and an anticipated non-response rate of 10%, the final sample size was estimated to be 1,400 children. Sample size estimation was performed using OpenEpi version 3.01 (Open Source Epidemiologic Statistics for Public Health, Atlanta, Georgia, USA).

Sampling and eligibility criteria

A two-stage proportionate cluster random sampling technique was employed. In the first stage, five schools were selected as clusters from the Nizamabad district using probability proportional to school enrollment. The selected clusters comprised two rural and three urban schools to ensure representation of both settings. In the second stage, eligible children were randomly selected from the class registers of each school in proportion to the number of enrolled students until the required sample size of 1,400 participants was achieved. Children aged 5-12 years with primary or mixed dentition, erupted maxillary and mandibular incisors, and erupted first permanent molars were included in the study. The requirement for erupted first permanent molars was necessary to permit a standardized assessment of molar relationships, one of the primary study variables. Children undergoing orthodontic treatment, those with craniofacial anomalies or systemic disorders affecting craniofacial growth, and those who were uncooperative during the clinical examination were excluded.

Calibration and data collection

Prior to data collection, the principal examiner underwent standardized training and calibration under the supervision of an experienced orthodontist to ensure consistency in the clinical assessment. The calibration involved repeated clinical examinations of children with a range of deep bite severities and standardized evaluation of all study variables. Any discrepancies identified during the calibration sessions were discussed and resolved by consensus before the commencement of the main survey. Subsequently, all clinical examinations were performed by the same calibrated examiner using a standardized examination protocol to minimize inter-observer variability.

A structured and pre-validated data collection form was developed to record demographic characteristics, oral habits, facial profile, body build, lower facial height, lip competence, mentolabial sulcus, upper labial frenal attachment, molar relationship, overjet, and deep bite severity (Appendix). The content validity of the proforma was assessed using the content validity ratio (CVR), and the final instrument achieved an acceptable CVR of 0.75 following expert evaluation.

Extraoral examination included assessment of body build, facial profile, lower facial height, lip competence, and mentolabial sulcus. Body build was assessed clinically during the general examination and categorized as aesthetic, athletic, or plethoric. An aesthetic body build was defined as a thin physique with relatively narrow body proportions; an athletic body build as a normally built, well-proportioned physique that was neither thin nor obese; and a plethoric body build as an obese or heavy-set physique with broad body proportions. The facial profile was assessed in the natural head position from the lateral view and classified as straight, convex, or concave according to the relative anteroposterior relationship of the maxilla and mandible. Lower facial height was evaluated clinically by assessing the proportion of the lower facial third and categorized as decreased, normal, or increased. Lip competence was assessed with the patient at rest and recorded as competent when the lips were in passive contact without muscular strain and incompetent when lip closure required muscular effort or the lips remained apart at rest. Mentolabial sulcus was evaluated visually and classified as normal, deep, or shallow according to its clinical depth and contour. Intraoral examination included assessment of the upper labial frenal attachment, molar relationship, overjet, and overbite.

Clinical examinations were performed under natural daylight using sterile mouth mirrors, community periodontal index (CPI) probes, and a digital Vernier caliper to assess overbite. Extraoral examination included assessment of body build, facial profile, lower facial height, lip competence, and mentolabial sulcus. In contrast, intraoral examination included evaluation of the upper labial frenal attachment, molar relationship, and overjet.

Overbite was measured using a digital Vernier caliper (accuracy: 0.01 mm) by recording the vertical overlap of the maxillary central incisors over the mandibular central incisors in maximum intercuspation. The measured overlap was then expressed as a percentage of the clinical crown height of the mandibular central incisor to standardize the assessment across children with varying tooth sizes. Deep bite was diagnosed clinically during the orthodontic examination. Following diagnosis, the severity of deep bite was categorized using a study-specific ordinal grading system based on the percentage of vertical overlap of the mandibular incisors with the maxillary incisors. This grading system was developed solely for epidemiological stratification of disease severity within the study population. It was intended to facilitate uniform statistical comparison rather than to establish diagnostic thresholds for deep bite. Deep bite severity was categorized as grade 1 (≤20% vertical overlap), grade 2 (>20-40%), grade 3 (>40-60%), and grade 4 (>60% vertical overlap). The grade 1 category represented the lowest category within the study-specific severity scale. It was retained to provide an ordinal framework for analysis across the full spectrum of overbite measurements rather than to denote a pathological deep bite threshold. The grading system was intended to provide a simple, reproducible method for epidemiological stratification of deep bite severity. It was applied consistently by a single calibrated examiner using a standardized clinical protocol. The modified classification has not undergone external validation and should therefore be interpreted as a study-specific epidemiological tool.

Statistical analysis

Data were then analyzed using SPSS Statistics version 25.0 (IBM Corp. Released 2017. IBM SPSS Statistics for Windows, Version 25.0. Armonk, NY). Descriptive statistics were expressed as frequencies and percentages. Associations between deep bite grades and categorical variables were evaluated using the chi-square test of independence or Fisher's exact test, and the strength of significant associations was quantified using Cramér's V effect size. Variables demonstrating statistical significance in the bivariate analysis were entered into a cumulative odds ordinal logistic regression model using the enter method to identify the independent predictors of deep bite severity. The proportional odds assumption was verified using a test of parallel lines before interpreting the regression model. Statistical significance was established at p < 0.05 for all analyses.

## Results

A total of 1,400 children were included in this study. Overall, 734 (52.4%) had grade 1 deep bite, 337 (24.1%) had grade 2, 259 (18.5%) had grade 3, and 70 (5.0%) had grade 4 deep bite. Table 1 shows the association between oral habits and deep bite severity. Most children (1,246, 89.0%) had no deleterious oral habits, whereas finger sucking was reported in 69 (4.9%), mouth breathing in 39 (2.8%), lip sucking in 20 (1.4%), nail biting in 17 (1.2%), and tongue thrusting in nine (0.7%). Oral habits were significantly associated with deep bite severity (p < 0.001, Cramér's V = 0.104); however, the effect size indicated that the association was weak, indicating limited clinical significance despite statistical significance.

**Table 1:** Association between deep bite severity and oral habits Data are presented as numbers (percentages); associations were analyzed using Fisher's exact test; effect size was assessed using Cramér's V; *p < 0.05 was considered statistically significant. Grade: deep bite grade

Oral habits	Grade 1	Grade 2	Grade 3	Grade 4	Total	Chi stats	p-value	Effect size
Never	668 (47.7%)	295 (21.1%)	224 (16.0%)	59 (4.3%)	1,246 (89.0%)	45.67 (15)	<0.001*	0.104
Mouth breathing	23 (1.6%)	9 (0.6%)	4 (0.3%)	3 (0.2%)	39 (2.8%)			
Lip sucking	5 (0.4%)	3 (0.2%)	9 (0.6%)	3 (0.2%)	20 (1.4%)			
Tongue thrusting	7 (0.5%)	0 (0.0%)	1 (0.1%)	1 (0.1%)	9 (0.7%)			
Finger sucking	17 (1.2%)	29 (2.1%)	21 (1.5%)	2 (0.1%)	69 (4.9%)			
Nail biting	14 (1.0%)	1 (0.1%)	0 (0.0%)	2 (0.1%)	17 (1.2%)			
Total	734 (52.4%)	337 (24.1%)	259 (18.5%)	70 (5.0%)	1,400 (100%)			

Body build and facial profiles were significantly associated with deep bite severity (Table 2). An athletic body build was the predominant type, observed in 1,000 children (71.4%), followed by an aesthetic body build in 205 (14.7%) and a plethoric body build in 195 (13.9%). Regarding facial profile, a straight profile was present in 819 (58.5%), followed by a convex profile in 507 (36.2%) and a concave profile in 74 (5.3%). Body build (χ² = 42.356, p < 0.001) and facial profile (χ² = 55.894, p < 0.001) were significantly associated with deep bite severity.

**Table 2:** Association between deep bite severity, body build, and facial profile Data are presented as counts (percentages); associations were analyzed using the chi-square test of independence; effect size was assessed using Cramér's V; and *p < 0.05 was considered statistically significant.

Parameter	Category	Grade 1	Grade 2	Grade 3	Grade 4	Total	Chi stats	p-value	Effect size
Body build	Aesthetic	108 (7.7%)	43 (3.1%)	45 (3.2%)	9 (0.7%)	205 (14.7%)	42.356 (df = 6)	<0.001*	0.124
	Plethoric	93 (6.6%)	74 (5.3%)	26 (1.9%)	2 (0.1%)	195 (13.9%)			
	Athletic	533 (38.1%)	220 (15.7%)	188 (13.4%)	59 (4.2%)	1,000 (71.4%)			
	Total	734 (52.4%)	337 (24.1%)	259 (18.5%)	70 (5.0%)	1,400 (100%)			
Facial profile	Concave	54 (3.9%)	13 (0.9%)	6 (0.4%)	1 (0.1%)	74 (5.3%)	55.894 (df = 6)	<0.001*	0.141
	Straight	499 (35.6%)	152 (10.9%)	136 (9.7%)	32 (2.3%)	819 (58.5%)			
	Convex	181 (12.9%)	172 (12.3%)	117 (8.4%)	37 (2.6%)	507 (36.2%)			
	Total	734 (52.4%)	337 (24.1%)	259 (18.5%)	70 (5.0%)	1,400 (100%)			

Table 3 summarizes the association between deep bite severity and the extraoral examination findings. Normal lower facial height was observed in 642 children (45.9%), decreased lower facial height in 462 (33.0%), and increased lower facial height in 296 (21.1%). Competent lips were present in 1,045 (74.6%), while 355 (25.4%) exhibited lip incompetence. A normal mentolabial sulcus was observed in 786 (56.1%), followed by a deep sulcus in 383 (27.4%) and a shallow sulcus in 231 (16.5%). Lower facial height (χ² = 238.765), lip competence (χ² = 27.891), and mentolabial sulcus (χ² = 39.452) were significantly associated with deep bite severity (p < 0.001).

**Table 3:** Association between deep bite severity and extraoral examination parameters Data are presented as numbers (percentages). Associations were analyzed using the chi-square test of independence. Effect size was assessed using Cramér's V. *p < 0.05 was considered statistically significant. LFH: lower facial height

Parameter	Category	Grade 1	Grade 2	Grade 3	Grade 4	Total	Chi stats	p-value	Effect size
Lower facial height	Increased LFH	194 (13.8%)	57 (4.1%)	30 (2.1%)	15 (1.1%)	296 (21.1%)	238.765 (df = 6)	<0.001*	0.292
	Decreased LFH	96 (6.9%)	164 (11.7%)	155 (11.1%)	47 (3.3%)	462 (33.0%)			
	Normal LFH	444 (31.7%)	116 (8.3%)	74 (5.3%)	8 (0.6%)	642 (45.9%)			
	Total	734 (52.4%)	337 (24.1%)	259 (18.5%)	70 (5.0%)	1,400 (100.0%)			
Lip competence	Competent	600 (42.8%)	218 (15.6%)	175 (12.5%)	52 (3.7%)	1,045 (74.6%)	27.891 (df = 3)	<0.001*	0.141
	Incompetent	134 (9.6%)	119 (8.5%)	84 (6.0%)	18 (1.3%)	355 (25.4%)			
	Total	734 (52.4%)	337 (24.1%)	259 (18.5%)	70 (5.0%)	1,400 (100%)			
Mentolabial sulcus	Normal	485 (34.6%)	147 (10.5%)	124 (8.8%)	30 (2.1%)	786 (56.1%)	39.452 (df = 6)	<0.001*	0.119
	Deep	122 (8.7%)	120 (8.6%)	102 (7.3%)	39 (2.8%)	383 (27.4%)			
	Shallow	127 (9.1%)	70 (5.0%)	33 (2.4%)	1 (0.1%)	231 (16.5%)			
	Total	734 (52.4%)	337 (24.1%)	259 (18.5%)	70 (5.0%)	1,400 (100.0%)			

As shown in Table 4, normal upper labial frenal attachment was observed in 1,318 children (94.1%), whereas high frenal attachment was present in 82 (5.9%). No significant association was found between upper labial frenal attachment and deep bite severity (χ² = 3.342, p = 0.341). Molar relationships were significantly associated with deep bite severity (χ² = 38.901; p < 0.001). Class I molar relationships were the most common, affecting 712 (50.9%), followed by Class II Division 1 in 318 (22.7%), mesial step in 246 (17.6%), distal step in 83 (5.9%), flush terminal plane in 18 (1.3%), Class II Division 2 in 13 (0.9%), and Class III in 10 (0.7%).

**Table 4:** Association between deep bite severity and intraoral examination parameters Data are presented as numbers (percentages); associations were analyzed using Fisher's exact test; effect size was assessed using Cramér's V; *p < 0.05 was considered statistically significant. Class I molar relationship: Mesiobuccal cusp of maxillary first molar occludes with mesiobuccal groove of mandibular first molar. Class II Div 1: Distobuccal cusp of maxillary first molar occludes with mesiobuccal groove of mandibular first molar, and proclined upper incisors. Class II Div 2: Distobuccal cusp of maxillary first molar occludes with mesiobuccal groove of mandibular first molar, and retroclined upper incisors. Class III: Mesiobuccal cusp of maxillary first molar occludes in the interdental space between mandibular first molar and second molar. Div: division

Parameter	Category	Grade 1	Grade 2	Grade 3	Grade 4	Total	Chi stats	p-value	Effect size
Upper labial frenal attachment	Normal	698 (49.8%)	311 (22.2%)	243 (17.4%)	66 (4.7%)	1,318 (94.1%)	3.342 (df = 3)	0.341	0.049
	High	36 (2.6%)	26 (1.9%)	16 (1.1%)	4 (0.3%)	82 (5.9%)			
	Total	734 (52.4%)	337 (24.1%)	259 (18.5%)	70 (5.0%)	1,400 (100%)			
Molar relationship	Mesial step	151 (10.8%)	40 (2.9%)	41 (2.9%)	14 (1.0%)	246 (17.6%)	38.901 (df = 18)	<0.001*	0.096
	Distal step	36 (2.6%)	24 (1.7%)	20 (1.4%)	3 (0.2%)	83 (5.9%)			
	Flush terminal plane	13 (0.9%)	2 (0.1%)	3 (0.2%)	0 (0.0%)	18 (1.3%)			
	Class I	408 (29.1%)	159 (11.4%)	110 (7.9%)	35 (2.5%)	712 (50.9%)			
	Class II Div 1	123 (8.8%)	101 (7.2%)	79 (5.6%)	15 (1.1%)	318 (22.7%)			
	Class II Div 2	7 (0.5%)	5 (0.4%)	1 (0.1%)	0 (0.0%)	13 (0.9%)			
	Class III	8 (0.6%)	1 (0.1%)	1 (0.1%)	0 (0.0%)	10 (0.7%)			
	Total	746 (53.2%)	332 (23.8%)	255 (18.2%)	67 (4.8%)	1,400 (100%)			

Ordinal logistic regression analysis (Table 5) demonstrated that athletic body build was an independent predictor of greater deep bite severity than aesthetic body build (OR = 6.505, p = 0.003). Increased lower facial height (OR = 3.362, p = 0.006), decreased lower facial height (OR = 9.966, p < 0.001), and increased overjet (OR = 1.255, p = 0.013) were also identified as significant independent predictors of greater deep bite severity. Age, sex, facial profile, lip competence, mentolabial sulcus, upper labial frenal attachment, and molar relationship were not independently associated with deep bite severity after multivariate adjustment.

**Table 5:** Cumulative odds ordinal logistic regression analysis for predictors of deep bite severity Data are presented as ORs (Exp(B)) and SE. Cumulative odds ordinal logistic regression using the enter method was performed to identify independent predictors of deep bite severity; the proportional odds assumption was verified using the test of parallel lines. *p < 0.05 was considered statistically significant; reference categories are indicated in parentheses. Grade 1: ≤20% vertical overlap, grade 2: >20-40% vertical overlap, grade 3: >40-60% vertical overlap, and grade 4: >60% vertical overlap. Exp(B): exponentiated regression coefficient, OR: odds ratio

Independent variable		Category 1 (grade 1 vs. 2 + 3 + 4)			Category 2 (grade 1 + 2 vs. 3 + 4)			Category 3 (grade 1 + 2 + 3 vs. 4)		
		SE	p-value	OR (95% CI)	SE	p-value	OR (95% CI)	SE	p-value	OR (95% CI)
Demographic	Age	0.210	0.323	1.231 (0.815-1.858)	0.219	0.102	1.431 (0.932-2.198)	0.373	0.376	0.719 (0.346-1.493)
	Gender	0.206	0.248	0.789 (0.527-1.181)	0.205	0.278	0.800 (0.535-1.195)	0.365	0.958	1.019 (0.498-2.083)
Body build (ref: aesthetic)	Plethoric	0.244	0.623	1.128 (0.699-1.818)	0.247	0.682	1.106 (0.682-1.795)	0.437	0.848	0.919 (0.390-2.167)
	Athletic	0.622	0.003*	6.505 (1.921-22.100)	0.695	0.255	2.206 (0.565-8.612)	1.148	0.353	2.907 (0.306-27.62)
Facial profile (ref: concave)	Straight	-	0.959	-	-	0.351	-	-	0.370	-
	Convex	0.544	0.774	0.855 (0.294-2.482)	0.578	0.578	0.725 (0.233-2.251)	0.483	0.997	1.110 (0.498-2.083)
Lower facial height (ref: normal	Increased	0.328	0.772	1.100 (0.578-2.092)	0.316	0.516	0.814 (0.438-1.511)	0.438	0.006*	3.362 (1.425-7.926)
	Decreased	0.291	0.088	1.643 (0.929-2.907)	0.315	0.796	1.085 (0.585-2.012)	0.562	0.000*	9.966 (3.313-29.94)
Lip (ref: competent)		0.340	0.099	0.571 (0.293-1.112)	0.319	0.392	1.315 (0.703-2.455)	0.576	0.228	2.004 (0.648-6.194)
Mentolabial sulcus (ref: normal)	Deep	0.300	0.227	1.437 (0.798-2.588)	0.283	0.458	1.234 (0.708-2.149)	0.452	0.347	0.654 (0.270-1.586)
	Shallow	0.293	0.329	1.331 (0.750-2.363)	0.314	0.111	1.650 (0.891-3.050)	0.646	0.661	1.328 (0.375-4.713)
Upper labial frenal attachment		0.395	0.182	0.590 (0.272-1.279)	0.391	0.695	0.858 (0.399-1.846)	0.620	0.395	0.591 (0.175-1.992)
Molar relation (ref: mesial step)	Class I	-	0.859	-	-	0.187	-	-	0.994	-
	Class II	0.271	0.745	1.092 (0.642-1.857)	0.257	0.069	0.627 (0.379-1.038)	0.480	0.915	0.950 (0.371-2.435)
	Class III	4.607	0.649	8.131 (1.872-28.118)	2.356	0.753	0.477 (0.205-1.435)	0.135	0.999	1.071 (0.598-2.183)
	Overjet	-	-	-	-	-	-	-	0.013*	1.255

## Discussion

The present cross-sectional epidemiological study evaluated the severity of deep bite and its associated demographic, extraoral, and intraoral factors among school-going children with primary and mixed dentition. A deep bite is one of the most common vertical malocclusions encountered during childhood, with previous epidemiological studies reporting prevalence estimates ranging from approximately 11.8% to 36.7% in different populations, depending on the diagnostic criteria, age group, and ethnic characteristics of the study population [8,9]. In the present study, grade 1 deep bite was the most frequently observed severity category, indicating that most affected children presented with relatively mild vertical malocclusion. These findings emphasize the importance of early orthodontic screening and timely interceptive treatment during the mixed dentition stage to prevent progression to more severe occlusal discrepancies.

A significant association was observed between deleterious oral habits and deep bite severity in the bivariate analysis. Habits such as finger sucking, lip sucking, mouth breathing, and tongue thrusting are well recognized for disrupting the balance between the tongue, lips, and buccal musculature, thereby influencing dentoalveolar development and occlusal relationships [8,9]. Previous epidemiological and observational studies have similarly demonstrated that prolonged oral habits contribute to the development of malocclusion during childhood [8,9]. However, these habits were not retained as independent predictors after multivariable adjustment in the present study. Moreover, finger sucking and tongue thrusting are more commonly associated with reduced overbite or anterior open bite than with deep bites. Therefore, the observed association should be interpreted cautiously and may reflect the influence of unmeasured craniofacial or dentoalveolar characteristics rather than a direct effect of oral habits on deep bite severity. This explanation is hypothetical and warrants confirmation in future longitudinal studies.

The most important findings of the present investigation were related to craniofacial morphology. Reduced lower facial height was significantly associated with greater deep bite severity in the bivariate analysis. Children with reduced lower facial height typically exhibit a hypodivergent facial pattern, increased masticatory muscle activity, and excessive vertical overlap of the incisors. These characteristics have consistently been associated with deep bite malocclusion [10,11]. These findings are consistent with the classical orthodontic concept that reduced anterior facial height is closely related to excessive overbite [4,11,12]. El-Dawlatly et al. [12] reported that vertical skeletal morphology is an important determinant of deep overbite. Similarly, Claro et al. [13] emphasized the importance of considering the vertical skeletal pattern during the diagnosis and management of deep bite. Beckmann et al. [14] further demonstrated that both skeletal and dentoalveolar factors contribute substantially to overbite development. Watted et al. [4] also emphasized that deep bite is a multifactorial malocclusion resulting from the interaction of skeletal, dentoalveolar, muscular, and environmental factors. However, the association between lower facial height and deep bite severity was not retained after multivariable adjustment in the present study, suggesting that its effect may be influenced by other correlated craniofacial characteristics rather than representing an independent predictor.

Increased overjet was also identified as an independent predictor of greater deep bite severity. Overjet was analyzed as a continuous variable (mm) in the regression model. An OR of 1.255 indicates that each 1-mm increase in overjet was associated with a 25.5% increase in the odds of exhibiting greater deep bite severity. However, this association was not retained after multivariable adjustment and should therefore be interpreted with caution. This finding supports previous reports suggesting that sagittal and vertical discrepancies frequently coexist during craniofacial development, particularly in children with Class II malocclusion [13]. Although increased overjet does not directly cause deep bite, both conditions often share common skeletal and dentoalveolar characteristics, including altered maxillomandibular relationships and abnormal incisor inclination. The coexistence of these features reinforces the importance of comprehensive orthodontic assessment during the mixed dentition stage.

Although body build, facial profile, lip competence, mentolabial sulcus, and molar relationship were significantly associated with deep bite severity in the bivariate analysis, only athletic body build remained an independent predictor after multivariable adjustment. To our knowledge, no previous epidemiological study has evaluated the association between body build and deep bite severity using multivariable analysis; therefore, direct comparison with the existing literature is not possible. The observed association may reflect differences in craniofacial growth patterns and masticatory muscle function rather than body build acting as an independent etiological factor. However, this finding should be interpreted cautiously, as body build was assessed using a clinical descriptive classification and the underlying biological mechanisms remain uncertain. Additionally, upper labial frenal attachment was not significantly associated with deep bite severity, suggesting that it is unlikely to contribute substantially to the development of vertical occlusal discrepancies.

The findings of the present investigation reinforce the multifactorial nature of deep bite and highlight the dominant contribution of skeletal growth characteristics over isolated intraoral findings [4,14]. Identification of children exhibiting reduced facial height and increased overjet during the mixed dentition stage may facilitate timely interceptive orthodontic intervention and minimize the need for more complex treatment during adolescence [15,16]. From a clinical perspective, these findings support the importance of routine orthodontic screening in school-aged children to facilitate the early identification of deep bites and associated craniofacial and occlusal characteristics. Early recognition may assist clinicians in comprehensive orthodontic assessment and appropriate treatment planning. However, given the cross-sectional design and the study-specific grading system, these findings should not be interpreted as evidence of disease progression or future treatment need. Further longitudinal studies are required to establish the prognostic value of the identified associations.

Limitations

Nevertheless, this study has certain limitations. Its cross-sectional design precluded causal or temporal inferences, and the findings should therefore be interpreted as associations rather than causation. Participants were recruited from a single district, which may limit generalizability. Craniofacial characteristics were assessed clinically without cephalometric evaluation, and the modified deep bite grading system was developed for epidemiological stratification and has not been externally validated. Additionally, body build was assessed using a clinical descriptive classification, detailed examiner reliability statistics were unavailable, and some regression estimates were imprecise because of sparse data in certain categories. Furthermore, the clustered sampling design was not explicitly accounted for in the regression analysis. Despite these limitations, the relatively large sample size, standardized clinical examination, and multivariable analysis strengthen the study. Future multicenter longitudinal studies incorporating objective anthropometric and cephalometric assessments are warranted to validate these findings.

## Conclusions

This study provides epidemiological data on the distribution of deep bite severity among children aged 5-12 years and demonstrates significant associations between deep bite severity and several craniofacial and occlusal characteristics. After multivariable analysis, only athletic body build remained significantly associated with greater deep bite severity; however, this novel finding should be interpreted cautiously because of the cross-sectional study design, the clinical descriptive classification of body build, and the lack of previous supporting evidence. The study-specific grading system was developed for epidemiological stratification and should not be considered a validated clinical severity classification. These findings support the importance of routine orthodontic screening and comprehensive clinical assessment in school-aged children. Further prospective studies are required to validate these associations and determine their clinical relevance.

## Appendices

**Table 6:** Study proforma

Section A. Demographic Details	
Variable	Response Options
Study ID	
Date of examination	
School name	
School type	□ Government □ Private
Village/area	
Age (years)	
Age group	□ 5-7 □ 8-10 □ 11-12
Sex	□ Male □ Female
Height (cm)	
Weight (kg)	
Section B. General History	
Feeding History	
Breastfeeding	□ Yes □ No
Duration of breastfeeding	□ <6 months □ 6-12 months □ >12 months
Bottle feeding	□ Yes □ No
Duration of bottle feeding	□ <1 year □ 1-2 years □ >2 years
Pacifier use	□ Yes □ No
Habit	
Mouth breathing	□ Yes □ No
Thumb/finger sucking	□ Yes □ No
Tongue thrusting	□ Yes □ No
Lip sucking	□ Yes □ No
Nail biting	□ Yes □ No
Bruxism	□ Yes □ No
Medical History	
Any systemic illness	□ Yes □ No
Previous orthodontic treatment	□ Yes □ No
Craniofacial anomalies	□ Yes □ No
History of facial trauma	□ Yes □ No
Section C. Extraoral Examination	
Body build	□ Aesthetic □ Athletic □ Plethoric
Facial profile	□ Straight □ Convex □ Concave
Facial divergence	□ Anterior □ Posterior □ Straight
Facial symmetry	□ Symmetrical □ Asymmetrical
Lower facial height	□ Increased □ Normal □ Decreased
Lip competency	□ Competent □ Incompetent
Mentolabial sulcus	□ Normal □ Deep □ Shallow
Chin prominence	□ Normal □ Prominent □ Retruded
Section D. Intraoral Examination	
Dentition	
Dentition stage	□ Primary □ Mixed
Upper labial frenal attachment	□ Normal □ High attachment
Overjet (mm)	
Overbite (mm)	
Deep bite (%)	
Molar Relationship	
Primary dentition	□ Flush terminal plane □ Mesial step □ Distal step
Permanent dentition	□ Class I □ Class II Div 1 □ Class II Div 2 □ Class III
Occlusion	
Crossbite	□ Yes □ No
Open bite	□ Yes □ No
Crowding	□ Yes □ No
Spacing	□ Yes □ No
Lower anterior attrition	□ Present □ Absent
Modified Deep Bite Index	
Grade	Description
Grade 1	□ Normal overbite
Grade 2	□ Mild deep bite
Grade 3	□ Moderate deep bite
Grade 4	□ Severe deep bite
